# Intrauterine Inflammation, Epigenetics, and Microbiome Influences on Preterm Infant Health

**DOI:** 10.1007/s40139-018-0159-9

**Published:** 2018-01-20

**Authors:** Lei Lu, Erika C. Claud

**Affiliations:** 1 Department of Pediatrics/Neonatology, University of Chicago, 900 E 57th Street, Chicago, IL 60637 USA; 2 Department of Pediatrics/Neonatology, University of Chicago, 5143 Maryland Street, Chicago, IL 60637 USA

**Keywords:** Preterm birth, Intrauterine infection, Perinatal immune response, Epigenetic modification, Early microbiota and development

## Abstract

**Purpose of Review:**

Significant research reveals that the microbiome modulates perinatal and postnatal health. This review aims to examine mechanisms by which intrauterine infection, the epigenome, and microbiome specifically influence preterm infant health outcomes.

**Recent Findings:**

Intrauterine infection is a primary cause of preterm birth and can cause alterations in gene expression and epigenetic programming as well as postnatal inflammatory responses in the offspring. Insights from our own studies demonstrate epigenetic modifications of TLRs associated with exposure to intrauterine inflammation, as well as a cross talk between host epigenome and microbiome. Lastly, the gut microbiome modulates maturation of inflammatory pathways, which influences the development of preterm infants.

**Summary:**

We present a unifying theme that preterm infant outcomes are associated with modulation of host immune and inflammatory responses, which are influenced by acute intrauterine infection, epigenetic, and microbiome factors.

## Introduction

Preterm birth (PTB), defined as birth at < 37 completed weeks gestation, is one of the leading obstetrical problems in the USA, affecting nearly one out of every nine infants born annually [1]. PTB is the greatest risk factor for infant death, contributing to 35% of all infant deaths and health-care costs in excess of US $26 billion annually [2]. Premature babies are at risk for a number of health complications such as poor growth and respiratory and gastrointestinal disorders, as well as long-term poor neurodevelopmental outcomes including cerebral palsy, developmental delay, cognitive difficulties, and visual and hearing impairments.

From a pathophysiological perspective, PTB is a highly complex and incompletely understood syndrome. There are a number of factors including uteroplacental ischemia, cervical disease, decidual hemorrhage, stress, infection, and inflammation in the initiation of PTB [3, 4]. Among the various factors that are associated with preterm labor, infection and inflammation represent significant primary causes. It is estimated that as many as 50% of preterm labor cases are associated with infection and/or inflammation within the gestational compartment, with a higher proportion at earlier gestational ages. Infection induces a cascade of molecular events, such as activation of the innate immune system, elevation of inflammatory cytokines, prostaglandins, and contraction-associated proteins. These events ultimately lead to end-organ phenomena characteristic of labor, including uterine contractions, cervical dilation, and rupture of fetal membranes [5]. However, the pathophysiological pathways of preterm birth are beyond the scope of this review; please refer to references [4, 6–8] for in-depth up-to-date reviews on this topic. This work will discuss the interaction of intrauterine inflammation, epigenetic alteration, and early microbial colonization in preterm infant health and development.

## Intrauterine Inflammation and Premature Neonatal Inflammatory Responses

Gestation has long been recognized as a critical period for programming the eventual adult phenotype [9]. Thus, it is a time when adverse in utero conditions can induce alterations in normal development programs leading to long-lasting deficits in the organs or systems affected [9]. One such adverse condition is the exposure to microorganisms leading to inflammatory responses in both maternal (chorionic) and fetal (amniotic) tissues [8]. Antenatal inflammatory responses to infectious stimuli may have positive or negative effects on normal fetal developmental programming and potentially influence host immune responses later in life [10•].

Inflammation is considered part of the normal signaling pathway for parturition at term, and a premature activation of this pathway may lead to premature labor. For example, in animal models of preterm labor, deliberate infection of mice with bacteria increases pro-inflammatory cytokines (IL-1 and TNF-alpha) and induces labor [11]. Various infections such as urinary tract infections, bacterial vaginosis, sexually transmitted infections, malaria, and periodontal disease have been associated with PTB [12]. Even subclinical intrauterine infections stimulate the release of pro-inflammatory proteins that overlap with the mechanism of normal parturition [8]. Intrauterine inflammation may present as chorioamnionitis. Chorioamnionitis is a clinical condition with maternal inflammatory responses which may lead to preterm rupture of membrane and preterm labor [13]. The fetuses exposed to chorioamnionitis may develop fetal inflammatory response syndrome either as a clinical systemic inflammatory response defined by a fetal plasma interleukin-6 level higher than 11 pg/mL, or as a subclinical funisitis and fetal vasculitis diagnosed histologically [13]. A typical cytokine profile characterizing the fetal immune responses consists of high levels of innate inflammatory cytokines IL-1β, IL-6, IL-23, TNF-α, and IL-10, as well as chemokines IL-8, MCP1, and RANTES [14]. These changes are thought to significantly increase the susceptibility of neonates to diseases that are inflammatory in nature. Recent studies examining the relationship between maternal and child inflammatory cytokine profiles postnatally demonstrated that maternal inflammatory cytokines levels (such as IL-10, TNF-α, IFN-γ, IL-5, IL-13), during pregnancy, have the greatest impact on the infants’ early cytokine levels in response to phytohemagglutinin stimulation at 2 months, with minimal influence from genetic factors [15]. Furthermore, Luciano et al. reported that prenatal exposure to inflammation and chorioamnionitis lead to changes in the peripheral regulatory T cell (Treg) pool in cord blood. This change might influence the role of Tregs in peripheral tolerance and the control of immune responses to pathogens [16]. These studies suggest possible cellular mechanisms by which maternal infection/inflammation during pregnancy elicits a reprogramming of the fetal innate as well as adaptive immune system.

Preterm newborns are particularly vulnerable as these potential altered immune responses occur in the context of immaturity of all organ systems. Much of the morbidity of preterm birth is inflammatory in nature including chronic lung disease [17], neonatal necrotizing enterocolitis (NEC) [18], and periventricular leukomalacia [19]. Recent studies have linked histological and clinical chorioamnionitis with increased risk of in-hospital morbidities, mortality, and neurodevelopmental impairment among extremely preterm neonates < 27 weeks [4]. In particular, NEC has been studied as a disease associated with prematurity and linked to immature regulation of inflammatory pathways. NEC is a devastating inflammatory bowel disease affecting approximately 10% of premature infants. It carries a mortality rate of up to 50% and is an independent risk factor for poor neurodevelopmental outcome. The two primary risk factors are prematurity and bacterial colonization [20, 21]. However, attempts to identify a single causative organism have been unsuccessful [22–26].

In our own studies, we used a modified animal model of in utero inflammation/preterm labor to investigate if intrauterine bacterial exposure might affect postnatal host responses to systemic inflammatory challenge. In this model, pregnant CD1 mice were injected in utero with either PBS or heat-killed *Escherichia coli* (HKE) at a dose that did not induce preterm birth despite prenatal inflammation. The 10-day-old offspring born from these dams was challenged with systemic administration of lipopolysaccharide (LPS, E.coli O55) and platelet activation factor (PAF). The combination of these pro-inflammatory agents has been shown to induce systemic inflammation leading to a NEC-like intestinal injury in animal models [27]. We then examined the systemic and tissue-specific inflammatory responses in these pups. LPS and PAF induced systemic inflammatory responses in both PBS-and HKE-exposed pups with elevated serum levels of the innate inflammatory cytokines IL-1α, IL-1β, IL-6, IL-12, IL-10, TNFα, MCP-1, and KC. Concomitantly, there were very low basal and induced levels of IL-2, 4, 5, 13, 17, and Foxp3. However, after LPS challenge, the pups exposed to HKE in utero exhibited significantly lower levels of IL-10 compared to PBS pre-exposed pups (*p* < 0.01). We also discovered differential gene expression patterns specifically in the small intestines of PBS- and HKE-pre-exposed pups. In response to LPS and PAF stimulation, there were differential expression patterns of major tight junction genes. Intestinal tight junctions seal adjacent epithelial cells together and form a barrier between the environment and the interior of the mammalian organism. Dysregulation of tight junction integrity and function plays a key role in the pathophysiology of diseases including NEC [28–30]. The core components of tight junctions include the claudins, occludin, and other cell adhesion proteins that interact with other cell surface proteins via their extracellular domains, while their intracellular domains interact with adaptor proteins such as actins, catenins, and other junction-interacting proteins [31, 32]. The adaptors recruit cytoskeleton components to the junction via protein kinase-mediated phosphorylation cascades [32]. In our in utero/neonatal inflammation model PBS-pre-exposed mouse ileum demonstrated upregulation of claudins: Cldn1, Cldn4, Cldn7, Cldn14; junction adhesion and interaction molecules: Mllt4, Mpdz, Inadl, Tjap1, and Igsf5. In contrast, the aforementioned genes were either downregulated or unchanged in HKE-pre-exposed mouse ileum. LPS/PAF further induced upregulation of Icam1, Actn2, and Ctnna1 and differentially inhibited expression of Tjap1, Magi3, Cldn11, Crb1, Cldn12, Tjp2, Smurf1, Tjp3, and Llgl1. These findings point to impaired tight junction formation and adaptation to withstand inflammatory challenge with in utero exposure to bacterial products. Innate immunity is a critical defense mechanism against pathogenic and inflammatory stimuli in the neonatal period. Even a mild adverse exposure to intrauterine inflammation without clinical indication of preterm labor and fetal demise can still induce reprogramming of normal immune system development and intestinal homeostasis during systemic inflammation in the offspring. The cellular and molecular mechanism of this pathophysiological change is unclear; the latency and persistency of the response in the offspring suggest the possibility of an underlying epigenetic mechanism at play. An epigenetic trait is a stably heritable phenotype resulting from changes in a chromosome without alteration in the DNA sequence [33]. There has been growing evidence that pathogens might manipulate epigenetic processes to influence host responses associated with immunity and inflammation. We will discuss this topic in the next section.

## Epigenetic Reprogramming of Host Genes in Response to Intrauterine Inflammation

Evidence supports the existence of a plastic interval during the prenatal and neonatal segments of life, in which a stable reprogramming of gene expression will occur and may predispose the individuals to future disease [34]. At a molecular level, epigenetic processes constitute a major mechanism by which environmental factors may establish a new phenotypic trait during this plastic interval [34, 35]. Three decades ago, Barker et al. coined the fetal origins of adult disease hypothesis that later became known as the developmental origin of adult disease hypotheses [36]. The unique characteristics of the fetal origin are (1) delayed onset of the insult phenotype until much later, (2) the adverse effect from fetal exposure can last a lifetime for the affected individual, and (3) there is often a genetic reprogramming resulting from the prenatal exposure [37]. Although extensive human epidemiological and animal model data support the hypothesis, the underlying cellular and molecular mechanisms are poorly understood. Recent animal model evidence demonstrates that transient prenatal environmental alterations influence the establishment of epigenetic gene regulation and that epigenetic dysregulation is implicated in human diseases [38]. These effects have the potential to affect neonatal as well as adult outcomes. DNA methylation is an epigenetic modification required for proper gene regulation and cellular differentiation during fetal development [39]. Subtle differences in the intrauterine environment may influence this tightly controlled process and result in epigenetic changes and stable phenotype differences [40].

Parets and colleagues have identified thousands of CG sites across the genome that are associated with preterm birth in a gestational age (GA)-dependent manner [41]. The associated genes are enriched for numerous developmental processes. For example, there are higher levels of DNA methylation in HDAC4 CG sites. HDAC4 possesses histone deacetylase activity and represses transcription when tethered to a promoter. Studies have shown that HDAC4 regulates bone and muscle development and promotes healthy vision [41]. There are several other GA-associated differential methylated CpG sites in DNMT1 (DNA methyltransferase 1), DNMT3A, DNMT3B, and the 5′UTR of TET1 (tet methylcytosine dioxygenase 1), which are genes that are involved in epigenetic regulation during development [41]. The mechanisms remain unclear, but inflammation may alter the appropriate maintenance of epigenetic profiles during pregnancy. For instance, bacterial infection has been shown to induce hypermethylation in the promoter region of the imprinted gene Igf2 in mice [42]. Igf2 is an imprinted gene that is implicated in placental and fetal development. These imprinted genes function as critical growth effectors and regulators of development [43••]. Liu et al. also reported an association between DNA methylation changes of the imprinted gene PLAGL1 (pleomorphic adenoma gene-like 1) and chorioamnionitis [44]. The authors examined the role of DNA methylation at multiple imprint regulatory regions (DMR) implicated in growth and development by types of PTB and infectious status and concluded that only in preterm infants with pathologically defined chorioamnionitis or funisitis is DNA methylation increased at the PLAGL1 DMR.

In our studies, we examined epigenetic regulation of 22 key genes in the TLR-signaling pathway. TLRs are pattern recognition receptors that play a key role in the innate immune response. TLRs are activated by pathogen-associated molecular patterns on the surface of bacteria and viruses, result in cytoplasmic activation and nuclear translocation of NF-κB or interferon regulator factors (IRFs), and lead to the activation of genes encoding pro-inflammatory factors. Our previous work has demonstrated increased activation of NF-κB in immature enterocytes leading to an exaggerated inflammatory response. NF-κB activation is an early step in the intestinal injury in NEC [45]. Studies in human tissue have shown elevated levels of toll-like receptor 2 and 4 (TLR2 and TLR4), in fetal cells that is further increased in NEC tissue [46], and studies utilizing TLR4 knockout mouse models have identified a key role of increased TLR4 in NEC [45–48]. It has been suggested that epigenetics could be one of the mechanisms that regulate TLR gene expression. In our studies, we investigated a potential link between in utero bacterial exposure and DNA methylation changes of key genes in the TLR-signaling pathway. We found that in the ileum, prenatal inflammatory exposure resulted in hypermethylation of the promoter regions for Tlr2, Irf1, Irf3 Il6ra, and Irak1 genes, and hypomethylation of Hsp70, Irf8, Cd14, and Fadd genes involved in TLR-signaling pathway. Concurrently, we also tested the expression profiles of 84 genes involved in the TLR-signaling pathway and found a correlation between methylation patterns and the expression profiles by real-time PCR. These data suggest bacteria-mediated tissue-specific epigenetic modifications to the host genes. In summary, recent evidence supports the hypothesis that pathogens might initiate or influence the epigenetic processes of host cells, leading to epigenetic reprogramming, particularly in the processes associated with immunity and inflammation, which have been specifically implicated in preterm infant outcomes.

## Perinatal and Prenatal Microbiota and Homeostasis of the Host

Microbial communities inhabiting the human host play important roles in maintaining health status, including reproduction and early life programing, which is particularly important in the context of preterm neonates. The gut microbial ecosystem is known to play a critical role in the overall health of individuals. For the preterm infant, this extends to a critical role in development. Microbiota provide stimuli necessary for an adequate developmental programming of epithelial barrier function, gut homeostasis, angiogenesis, and innate and adaptive immune functions. Recent animal studies have demonstrated that the early neonatal period is critical for reaching microbiota-induced host homeostasis [49, 50]. Preterm infants have an altered developmental progression of microbial communities.

First, as noted above, preterm birth is often the result of a microbial dysbiosis or infection. Second, unique clinical features such as mode of delivery, perinatal antibiotic usage, delayed enteral feeding, and prolonged hospital stay also influence the preterm infant microbiome. Compared to the microbiome of full-term infants, preterm infants’ microbiota is characterized by lower diversity, more fluctuations, a higher representation of potential pathogenic microorganisms typically encountered in hospital environment, and a reduced representation of health-related commensal microorganisms [51, 52•].

Third, a dynamic cross talk exists between the epigenome of host and the initial colonizing microbiota such that altered epigenetic profiles can influence the initial colonizing bacteria. We experimentally found that antenatal dexamethasone treatment, clinically used in the management of women in preterm labor, induced alterations in epigenetic and gene expression profiles of TLR- and TJ-signaling pathways in the small intestine of the offspring. These changes were accompanied by changes in the microbiota composition at different taxonomic levels in the offspring at 2 weeks of life. At species level, we detected the biggest changes within the genus *Clostridium*, with an increase *C*. *aminophilum* (14.5 fold change enrichment (FCE)), *C*. *hathewayi* (1.42 FCE), and *C*. *orbisciendens* (1.20 FCE), as well as a decrease in *C*. *cocleatum* (− 11.96 FCE) and *C. lactatifermentans* (− 8.78 FCE) [53•].

Last, organ immaturity contributes to responses to and establishment of the microbiome in preterm infants. As previously noted, the primary risk factors for NEC are intestinal immaturity and bacterial colonization; thus, much research on the microbiome and preterm infants has focused on the role of the microbiome in NEC. Bacteria have always been linked to NEC. Antibiotics demonstrate treatment benefit and clinical studies have noted protection with breast feeding, increased risk with early life antibiotics, and prevention with probiotics [54–59]. Each of these interventions likely influences the intestinal microbiota. However, a specific pathogen has not been found to be associated with NEC. Consistent with the work of other investigators, our previous study demonstrated a difference in overall microbial community between NEC and control patients [60, 61]. We found decreased diversity, a bloom in *Proteobacteria* (specifically *Gammaproteobacteria*), and a decrease in *Firmicutes* prior to the clinical onset of NEC. Understanding the role of the early microbiota in development may be critical to explaining why some preterm infants do not develop NEC and to generating protective interventions for NEC.

However, alterations in the microbiome are not just relevant to preterm infant disease, but also to preterm infant growth, health, and normal development. A large body of literature demonstrates that in the gut, certain commensal bacteria play critical beneficial roles, including provision of essential nutrients, competitive colonization against pathogens, and intestinal maturation [62–67]. Recently, we described distinctive patterns of host microbiota interaction in a humanized gnotobiotic mouse model of gut development. In these studies, we transfaunated pregnant mice with early fecal microbiota from preterm infants (less than 2-weeks of life) with either low (M_PI_-L) or high (M_PI_-H) growth rates, as growth is a surrogate for health in this patient population. Microbiome analysis of M_PI_-L and M_PI_-H pups revealed different compositions reflecting the input human preterm infant microbiota dominated by *Proteobacteria* and *Firmicutes* in both M_PI_-L- and M_PI_-H-colonized mice. There was a greater contribution of *Bacteroidetes* (8.30 vs. 3.42) and *Actinobacteria* (8.53 vs. 0.57) in M_PI_-H-colonized mice compared to M_PI_-L mice [68••]. We found that the transfaunated mouse offspring exhibited the original preterm infants’ growth phenotype with the M_PI_-H pups exhibiting a higher growth rate and advanced maturation of the intestine. Intestinal samples of M_PI_-H pups revealed a mucosal surface with well-organized tight junctions, higher goblet and Paneth cell numbers, and higher expression of intestinal epithelial maturation marker gene Lgr5 [69••]. In contrast, the M_PI_-L offspring exhibited slower weight gain and poor development of the intestines represented by shorter villus height, lower proliferation in the crypts, and higher apoptosis rate [69••]. More strikingly, there was a constitutive overexpression of pro-inflammatory markers such as MCP1, VCAM, and nuclear translocation of activated NF-κB in the intestinal mucosa and elevated systemic pro-inflammatory cytokine levels in the serum of the M_PI_-L pups. Moreover, microarray analysis revealed very different expression profiles between M_PI_-L and M_PI_-H. The majority of upregulated genes in the small intestines of M_PI_-L pups were enriched in inflammatory processes. Thus, differences in the host microbiome influence development, growth, and control of pro-inflammatory responses in preterm infants.

## Conclusion and Future Perspectives

The health of preterm infants begins in utero. Maternal infection/inflammatory states during pregnancy can be deleterious to fetal and postnatal development and increase risk for preterm delivery. These deleterious effects involve alterations in gene expression and epigenetic programming that converge to induce inflammation, impair the immune system, and cause a series of pathologic conditions in preterm infants. In utero inflammation is further associated with postnatal inflammatory responses in the offspring associated with epigenetic modifications of TLRs associated with intrauterine exposure. Epigenetic modifications in TLRs are associated with differences in early microbial colonization patterns, and these early microbial patterns affect developmental outcomes for these vulnerable preterm infants.

More broadly, these recent scientific advances demonstrate that the human-associated microbiome exerts critical roles in prenatal and perinatal health, which could have a significant impact in future adulthood health. These microbiome colonization patterns can be influenced by maternal behavior, nutrition, and environmental factors. Recent laboratory studies have explored the reversibility of induced phenotypic effects and whether aberrant phenotypes induced in utero or during early development can be rescued [70, 71]. These studies suggested that some epigenetic signatures respond to changes in environment, are potentially reversible, and can be targeted for disease therapies. Research exploring methods of restoring aberrant phenotypes, either epigenetic or microbiome based, could lead to means of identifying susceptible individuals using screening for biomarkers during early life resulting in personalized health interventions with a goal of disease prevention, health, and wellness.
